# Is autism a disease of the cerebellum? An integration of clinical and pre-clinical research

**DOI:** 10.3389/fnsys.2013.00015

**Published:** 2013-05-10

**Authors:** Tiffany D. Rogers, Eric McKimm, Price E. Dickson, Dan Goldowitz, Charles D. Blaha, Guy Mittleman

**Affiliations:** ^1^Department of Psychology, The University of MemphisMemphis, TN, USA; ^2^Centre for Molecular Medicine and Therapeutics, Department of Medical Genetics, University of British ColumbiaVancouver, BC, Canada

**Keywords:** autism, cerebellum

## Abstract

Autism spectrum disorders are a group of neurodevelopmental disorders characterized by deficits in social skills and communication, stereotyped and repetitive behavior, and a range of deficits in cognitive function. While the etiology of autism is unknown, current research indicates that abnormalities of the cerebellum, now believed to be involved in cognitive function and the prefrontal cortex (PFC), are associated with autism. The current paper proposes that impaired cerebello-cortical circuitry could, at least in part, underlie autistic symptoms. The use of animal models that allow for manipulation of genetic and environmental influences are an effective means of elucidating both distal and proximal etiological factors in autism and their potential impact on cerebello-cortical circuitry. Some existing rodent models of autism, as well as some models not previously applied to the study of the disorder, display cerebellar and behavioral abnormalities that parallel those commonly seen in autistic patients. The novel findings produced from research utilizing rodent models could provide a better understanding of the neurochemical and behavioral impact of changes in cerebello-cortical circuitry in autism.

## Introduction

### Autism spectrum disorders (ASD)

Autism spectrum disorders are a group of neurodevelopmental disorders that have traditionally included autism, Asperger's syndrome, Rett syndrome, childhood disintegrative disorder, and pervasive developmental disorder not otherwise specified (American Psychiatric Association, 2000). With the impending arrival of the DSM-V, *Asperger's* syndrome, pervasive developmental disorder and childhood disintegrative disorder have been consolidated into the overarching category of ASD, while Rett syndrome has been removed from the diagnostic manual (Lauritsen, 2013). Disorders within the ASD spectrum are characterized by deficits in social skills and communication, stereotyped and repetitive behavior, and a range of cognitive function deficits that are diagnosed on average by 4.8 years of life, although symptoms suggestive of autism have been observed as early as 6 months of age (Rogers and DiLalla, 1990; American Psychiatric Association, 2000; Centers for Disease Control and Prevention, 2007; Ozonoff et al., 2010; Elsabbagh et al., 2012; Wolff et al., 2012). Deficits in cognitive function most commonly observed in ASD patients include impairments in memory and attention as well as impairments in executive function including planning, cognitive flexibility, rule acquisition, and abstract thinking (Ozonoff et al., 2007).

### The etiology of autism

The etiology of autism is multifaceted. Genetic factors are known to contribute to this disorder. According to studies investigating concordance rates in twins, the heritability of autism is approximately 60–90% (Bailey et al., 1995; Hallmayer et al., 2011). Studies exploring the contribution of genetics have revealed that multiple genes are likely contributors to this disorder (Muhle et al., 2004). Cytogenetic studies have found chromosomal abnormalities in regions such as 15q and 7q in ASD individuals (Muhle et al., 2004; Campbell et al., 2006; Schanen, 2006; Dimitropoulos and Schultz, 2007). Variations within these chromosomal loci are associated with an increased diagnosis of autism (Campbell et al., 2006). Whole-genome scans implicate chromosomes 1, 2, 4, 7, 10, 13, 15, 16, 17, 19, 22, and X (Muhle et al., 2004). Cytogenetic studies and whole-genome searches have yielded several candidate genes to be further researched (Muhle et al., 2004). However, all the currently known genetic variations contributing to autism only account for approximately 5–15% of cases (Devlin and Scherer, 2012). It is therefore likely that genetic factors alone are not sufficient to explain the etiology of autism.

Prenatal or perinatal environmental insults are also believed to contribute to autism. The developing brain is particularly susceptible to environmental injury early in development (Rice and Barone, 2000). Prenatal or neonatal exposure to certain chemicals, such as ethyl alcohol, produces neuropsychological deficits similar to autism, and several environmental causes have recently been shown specifically to result in both autistic symptoms and an increased likelihood of a diagnosis of autism (Landrigan, 2010). These chemicals include thalidomide, misoprostol, and valproic acid (VPA) (Moore et al., 2000; Rodier, 2002; Bandim et al., 2003). However, similar to genetic explanations of autism, exposure to known environmental agents accounts for a relatively small percentage of cases (Landrigan, 2010). Therefore, it is possible that autism results from a specific and as yet unknown combination of genetic predisposition and environmental insults with variation in these causes determining the severity of the phenotype.

Despite the current gap in understanding the etiology of autism, abnormalities in neuroanatomy, behavior, and the underlying genetics of patients diagnosed with autism may provide some insight into the causes of autistic symptomology. The aim of the current review is to suggest that accumulating clinical and preclinical research indicates that, regardless of etiology, developmental pathology of the cerebellum likely plays a very important role in autism and ASD. This hypothesis may provide a unifying framework for understanding the diversity of autism research findings as well as providing direction in the search for etiological factors in this disease.

## Evidence that cerebellum is involved in autism and ASD

Although traditionally implicated in motor function, accumulating evidence indicates that the cerebellum is also involved in cognitive function (Schmahmann and Caplan, 2006). The cerebellum is structurally and functionally abnormal in patients diagnosed with autism or within the ASD spectrum (Fatemi et al., 2012). Syndromes that share cognitive symptomology with autism also frequently share genetic mutations associated with abnormal cerebellar development.

### Structural cerebellar abnormalities are common in autism and ASD

Cerebellar neuropathology commonly occurs in ASD individuals. Cerebellar hypoplasia and reduced cerebellar Purkinje cell numbers are the most consistent neuropathologies linked to autism (Courchesne et al., 1988, 1994; Bauman, 1991; Courchesne, 1997; Palmen et al., 2004; DiCicco-Bloom et al., 2006). MRI studies report that autistic children have smaller cerebellar vermal volume as compared to typically developing children (Webb et al., 2009). Postmortem studies report that in addition to reduced Purkinje cell numbers, microanatomic abnormalities of the cerebellum in this population include excess Bergmann glia, reductions in the size and number of cells in the cerebellar nuclei, and an active neuroinflammatory process within cerebellar white matter (Bailey et al., 1998; Bauman and Kemper, 2005; Vargas et al., 2005).

Multiple disorders within the autism spectrum are also associated with cerebellar abnormalities. For example, Asperger's syndrome has been associated with lower total cerebellar volume and lower gray matter volume in the right cerebellum (McKelvey et al., 1995; Hallahan et al., 2009; Yu et al., 2011). Diffusion tensor magnetic resonance imaging has demonstrated that adults with Asperger's syndrome have reduced fractional anisotropy in the cortico-cortical parallel fibers and Purkinje cell fibers of the cerebellum and superior cerebellar peduncle of the right hemisphere, which indicates abnormal microstructure of cerebellar white matter. According to postmortem studies, patients with Rett syndrome display reduced volume of the cerebellum, cerebellar atrophy, and reduced Purkinje cell numbers (Oldfors et al., 1990; Murakami et al., 1992). Diffusion tensor imaging of cerebellar pathways in children with ASD found increased diffusivity of the superior cerebellar peduncles, suggesting abnormal connectivity between the cerebellum and the rostral-going cerebellar projections (Sivaswamy et al., 2010).

### Abnormalities of cerebellar function in autism and ASD are associated with deficits in cognitive and motor behavior, and social reward

Imaging studies indicate that in addition to neuropathological changes, the cerebellum is functionally abnormal in ASD patients. Functional MRI (fMRI) work has revealed that during a visually guided saccade task, ASD patients have increased activation of cerebello-thalamic circuitry compared to controls (Takarae et al., 2007). It has been reported that activation of the ipsilateral anterior cerebellar hemisphere is increased in magnitude and larger in spatial extent during simple motor tasks in ASD patients as compared to control participants (Allen et al., 2004). In adults with Asperger's syndrome, diffusion tensor magnetic resonance imaging showed that the level of severity of social impairment was significantly negatively correlated with the degree of anisotropy of the superior cerebellar peduncle, the primary output path of the cerebellum. This suggests that as cerebellar white matter decreases, symptom severity increases (Catani et al., 2008).

ASD individuals and individuals with cerebellar abnormalities occurring during development or following acute injury have similar cognitive impairments, suggesting that the cerebellum is directly involved in autistic symptoms. Clinical studies have shown that progressive cerebellar atrophy as well as acute cerebellar lesions caused by tumors or infarction result in impairments to attention, planning, set-shifting, reversal learning, verbal fluency, abstract reasoning, memory, and associative learning (Bracke-Tolkmitt et al., 1989; Botez-Marquard and Botez, 1993; Courchesne et al., 1994; Schmahmann and Sherman, 1998; Thoma et al., 2008; Baillieux et al., 2010). In individuals with developmental reductions in cerebellar volume, cognitive functions such as associative learning, verbal ability, planning, and working memory are impaired, and the degree of volume reduction is correlated with the degree of cognitive impairment (Steinlin, 2008; Bolduc et al., 2011, 2012).

In addition to cognitive deficits, individuals with autism exhibit motor deficits [reviewed in Gowen and Hamilton (2013)] which begin in infancy (Provost et al., 2007; Brian et al., 2008) and continue through adolescence and into adulthood (Fournier et al., 2010; Van Waelvelde et al., 2010). Motor deficits appear to be relatively prevalent in this patient group, having been observed in 21–100% of individuals with autism, depending on the sample (Ghaziuddin et al., 1994; Manjiviona and Prior, 1995; Miyahara et al., 1997; Green et al., 2002; Pan et al., 2009). Individuals with autism exhibit both gross and fine motor deficits including slow and repetitive hand and foot movements (Dowell et al., 2009), slow and inaccurate manual dexterity (Green et al., 2002), unstable balance (Freitag et al., 2007), and impaired gait (Jansiewicz et al., 2006). Interestingly, motor deficits and cognitive deficits appear to be related in autism. Specifically, better motor skills predict better daily living skills (Jasmin et al., 2009) and deficits in motor control predict increased severity of autistic symptoms in adulthood (Sutera et al., 2007). The relationship between autism and motor deficits is unsurprising considering that (1) cerebellar Purkinje cell loss is consistently reported in autism (Fatemi et al., 2012) and (2) the cerebellum is able to affect regions which control cognitive and motor functions via reciprocal connections to prefrontal cortex (PFC), posterior parietal cortex, and cortical motor regions (Strick et al., 2009).

The mechanisms through which cerebellar deficits may affect motor function in autism are currently unknown. Considering that the cerebellum is an important region in the development of internal models of action (i.e., models which predict the sensory consequences of motor commands and adapt to errors), one possibility is that motor deficits in autism result from impairments in this ability. Haswell et al. (2009) observed children with autism and age-matched controls as they learned to maneuver a robotic arm. These authors reported that children with autism built a stronger than normal association between self-generated motor commands and proprioceptive feedback, suggesting that individuals with autism process and integrate motor information differently than controls. Interestingly, these authors also found a strong positive relationship between the reliance on proprioception and impairments in social function, suggesting the possibility of a relationship between cerebellar pathology and motor and cognitive function in autism.

However, the relationship between autism and impairments in the formation or use of internal models of action has not consistently been found. Gidley Larson et al. (2008) tested children with autism on a prism adaptation task and a reaching task to assess their ability, relative to controls, to form internal models of action. In the prism adaptation task, children were asked to throw balls to visual targets with and without prism goggles which shifted the map of the visual environment. In the reaching task, children were required to move the handle of a robotic arm which imposed forces on the hand or displaced the cursor associated with the handle position. In both tasks, children with autism performed indistinguishably from controls, suggesting that motor impairments seen in autism do not result from impaired ability to form or use internal models of action. These results are similar to those of Mostofsky et al. (2004) who found no performance differences between children with high functioning autism and controls on a catching task known to depend on the cerebellum. Thus, although it appears that motor deficits in autism may be related to an underlying deficit in the ability to form internal models of action, the factors which modulate this relationship are not yet clear.

It should also be noted that individuals diagnosed with ASD also exhibit impairments in reward processing [reviewed in Dichter and Adolphs (2012)]. Learning studies indicate that this deficit appears to be specific to social as opposed to non-social rewards. For example, Lin et al. (2012) compared performance of individuals with and without autism on an instrumental reward learning task in which the reward was social (pictures of positive and negative faces) or non-social (winning or losing money). Individuals with autism exhibited a specific behavioral insensitivity to social rewards. In addition to learning studies, autonomic response studies also indicate that individuals with autism are impaired in social reward processing. For example, Sepeta et al. (2012) examined the pupillary response in children with and without autism and found that, in comparison to controls, children with autism exhibited decreased pupillary diameter when looking at happy faces. These authors interpreted these differential findings as indicative of reduced sensitivity to reward value of social stimuli in children with autism. Gaze orientation studies in which children with autism fail to orient to naturally occurring social stimuli also provide evidence of impaired social reward processing in autism (Dawson et al., 1998; Klin et al., 2009). Neuroimaging studies indicate that differences in reward circuitry likely underlie the observed differential performance of controls and children with autism on behavioral tasks (Dichter et al., 2012a,b). In support of the hypothesis that the reward impairment in autism is specific to social stimuli, Cascio et al. (2012) found similar responses of neural reward regions to food cues in children with autism and typically developing controls. Collectively, these studies suggest that impaired social skills in autism may result from reduced feelings of reward in response to social stimuli.

Involvement of the cerebellum in deficits in social reward has not been investigated in humans. It is well known, however, that disruption of afferents to the cerebellum results in deficits in the acquisition of classical eyeblink conditioning and causes extinction of eyeblink conditioning in well trained animals (McCormick et al., 1985; McCormick, 2005). Most recently it has been reported that temporary, bilateral inactivation of the cerebellar dentate nuclei reduced operant responding for a food reward (Peterson et al., 2012). These studies are therefore consistent with the idea that the cerebellum potentially may play an important role in human reward processing.

### Multiple genes necessary for normal cerebellar development are associated with the symptomology of autism

Although single genes have diverse influences on brain development, mutations of several genes that contribute to normal cerebellar development are consistently associated with increased susceptibility to autism. These genes include the retinoic acid-related orphan receptor alpha (RORα), Engrailed 2 (EN2), Ca^2+^-dependent activator protein for secretion 2 (CAPS2), and mesenchymal-epithelial transition (MET) receptor tyrosine kinase (Campbell et al., 2006; Wang et al., 2008; Yang et al., 2008; Sadakata and Furuichi, 2009; Nguyen et al., 2010; Sen et al., 2010). RORα is a gene required for differentiation of cerebellar Purkinje cells (Boukhtouche et al., 2010). The EN2 gene has been associated with normal cerebellar development, and mutations or deletions of EN2 result in reduced volume in the cerebellum and foliation abnormalities (Joyner, 1996; Kuemerle et al., 2007). CAPS2 contributes to normal cerebellar development by enhancing release of brain-derived neurotrophic factor (BDNF) and neurotrophin-3 (NT-3) (Sato et al., 2008; Sadakata and Furuichi, 2009). The MET receptor gene has been found to contribute to pre- and post-natal cerebellar proliferation as well as migration of cerebrocortical cells (Powell et al., 2001, 2003; Ieraci et al., 2002).

Genetic mutations associated with increased risk for syndromes associated with autistic symptomology are also associated with abnormal cerebellar development. Patients with fragile X syndrome, which results from a mutation of the fragile X mental retardation 1 (FMR1) gene, exhibit cognitive symptoms similar to autism such as hypersensitivity to auditory, tactile, gustatory, and olfactory stimuli, deficits in attention, mental disfunction, and perseveration (Jin and Warren, 2000; Tsiouris and Brown, 2004). Cerebellar abnormalities such as ectopic Purkinje cells, focal cerebellar Purkinje cell loss, and Bergmann gliosis are associated with fragile X syndrome (Sabaratnam, 2000; Greco et al., 2011). Mutations of the methyl CpG-binding protein 2 (MECP2) gene are known to produce Rett syndrome, a disorder within the autism spectrum characterized by language impairments, motor impairments, and stereotypical behavior (Amir et al., 1999). Patients with Rett syndrome frequently have cerebellar atrophy that increases with age (Murakami et al., 1992).

Multiple environmental agents, including VPA, an antiepileptic drug, and chlorpyrifos, an organophosphate pesticide, have been associated with an increased risk of autism (Moore et al., 2000; de Cock et al., 2012). Basic research also indicates that these agents interrupt normal cerebellar development. Yochum et al. (2008) report that rats prenatally exposed to VPA exhibit an increased number of apoptotic cells in the cerebellum. Ingram et al. (2000) also showed that prenatal exposure to VPA in rats results in a decrease of Purkinje cells. Krishnan et al. (2012) showed that in both young and adult mice, dermal exposure to chlorpyrifos resulted in increased glial fibrillary acidic protein expression of the cerebellum, suggesting neurotoxic effects in the cerebellums of these mice. Cole et al. (2011) reported that repeated postnatal exposure to chlorpyrifos resulted in changes in cerebellar gene expression. Abou-Donia et al. (2006) also reported that rats prenatally exposed to chlorpyrifos had fewer Purkinje cells at postnatal day 90.

It is also worth noting that an association between maternal fever during pregnancy and the risk of ASD has recently been reported. Zerbo et al. (2013) compared the incidence of maternal fever during pregnancy in children with ASD, developmental delay (DD), or typically developing controls. It was found that neither ASD or DD were associated with maternal influenza, but both conditions were associated with maternal fever during pregnancy. Further, the risk of ASD was reduced in mothers who also reported taking fever reducing medications, in comparison to mothers who did not. Although mechanisms underlying the association between maternal fever and ASD have not been investigated, it should be remembered that the cerebellum is one brain structure that has a very prolonged developmental time course that extends in humans from early embryogenesis until the first postnatal years (ten Donkelaar et al., 2003). It has been suggested that this protracted development makes the cerebellum uniquely vulnerable to a broad spectrum of developmental disorders (ten Donkelaar et al., 2003). The cerebellum may also be differentially vulnerable to fever. Preclinical research indicates that hyperthermic conditions induce apoptosis, disrupt neuronal maturation and result in an enhanced activation of heat shock proteins in the developing cerebellum (Khan and Brown, 2002; Maroni et al., 2003; Aydin et al., 2011; Dean et al., 2012).

## Autism disconnection hypothesis

Together, the recent findings implicating the cerebellum in autism suggest that by genetic and/or environmental causes, neuropathological changes in the cerebellum occur in individuals with ASD. These changes may then result in a loss or dysregulation of cerebellar output leading to a cascade of events which either directly or indirectly results in the behavioral and cognitive symptoms associated with the diagnosis of ASD. Thus, the autism disconnection hypothesis presented here posits that a disconnection in the autistic brain occurs as a result of developmental cerebellar neuropathology and particularly the loss of Purkinje cells in the cerebellar cortex as these cells constitute the outflow of cerebellar cortex (Palmen et al., 2004; Amaral et al., 2008; Whitney et al., 2008).

The disconnection hypothesis asserts that the etiology of a given disorder can be accounted for by a loss of connectivity between two or more brain areas (Catani and Ffytche, 2008). While this theory was originally used to explain literal severances of circuitry, this hypothesis has more recently been expanded to include developmental neuropathology or disturbance or dysregulation of neural circuitry resulting in impaired functional connectivity (Geschwind, 1965; Geshwind and Levitt, 2007). A disconnection occurring due to a change in cerebellar output in ASD patients could result not only in impaired communication between the cerebellum and its efferent targets, but also in compensations or adaptations of affiliated neuronal circuitry.

It is possible that a disconnection of cerebello-cortical projections could account for many of the cognitive symptoms of autism. Middleton and Strick (2000) used retrograde transportation of herpes simplex virus to trace multiple closed-loop, basal ganglia- and cerebello-cortical circuits. Notably, this study reported that the cerebellum projects, via the thalamus, to areas 46 and 9 of the PFC which is known to be involved in cognitive functions such as memory, planning, and decision making (Middleton and Strick, 2000; Schmahmann, 2001). Research also provides a second cerebellum-PFC circuit that could influence cognitive functions commonly attributed to the frontal cortex. The cerebellum projects indirectly to the ventral tegmental area (VTA) which contains dopamine cell bodies comprising the mesocortical dopaminergic system that terminates in the PFC (Fallon and Moore, 1978). Abundant evidence links PFC dopaminergic function to a number of important cognitive abilities including working memory, attentional selection, and cognitive flexibility (Robbins and Roberts, 2007). Therefore, a loss of cerebellar output could result in significant downstream effects on either of these cerebello-PFC circuits that, in turn, could result in deficits in higher cognitive functions that commonly occur in ASD.

**Figure 1:**
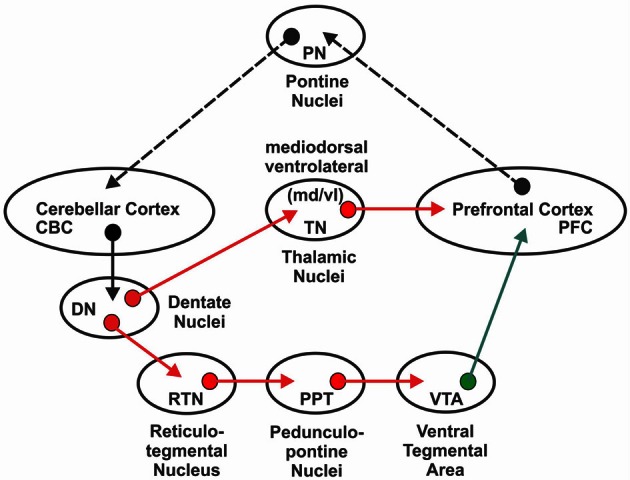
**Neural circuitry involved in cerebellar modulation of medial prefrontal cortex dopamine proposed to be affected by a developmental disconnection in autism.** With the exception of inhibitory cerebellar to dentate nucleus projections, red arrows indicate glutamatergic pathways. The green arrow indicates the mesocortical dopaminergic pathway. Dotted black arrow indicates feedback loop. See text for additional details and references.

The proposed neuronal circuitry, based on these findings, by which the cerebellum modulates PFC dopamine release is illustrated in Figure 1. Both of these neuronal circuits originate in cerebellar cortex Purkinje neurons that project to the deep cerebellar nuclei including cerebellar dentate nucleus (DN). The cerebello-ventral tegmental-cortical circuit involves indirect activation of mesocortical dopaminergic neurons via contralateral projections of the DN to reticulo-tegmental nuclei (RTN) that, in turn, project to pedunculopontine nuclei (PPT) and then project to, and stimulate directly, VTA dopaminergic cell bodies projecting to the medial PFC (mPFC) (Snider et al., 1976; Perciavalle et al., 1989; Schwarz and Schmitz, 1997; Garcia-Rill et al., 2001; Forster and Blaha, 2003; Mittleman et al., 2008; Rogers et al., 2011). The cerebello-thalamocortical circuit involves activation of the contralateral projections of the DN to thalamic mediodorsal/ventrolateral nuclei (ThN md/vl) that send afferents to the mPFC to modulate mesocortical dopaminergic terminal release in the mPFC via appositional excitatory glutamatergic synapses (Middleton and Strick, 2001; Pinto et al., 2003; Del Arco and Mora, 2005; Mittleman et al., 2008). Considered together, this anatomical connectivity suggests that changes in either or both of these circuits could result in aberrant dopaminergic activity in the mPFC.

If developmental cerebellar damage affects PFC processing, it should be expected that abnormalities in the target structure (i.e., PFC), as well as at the level of the neural junctions within these circuits (i.e., thalamus and VTA), should occur in ASD individuals. As previously discussed, several cerebellar abnormalities are affiliated with autism, but recent research also suggests that the PFC and thalamus are abnormal in these individuals (Tsatsanis et al., 2003; Carper and Courchesne, 2005). While abnormalities of the VTA in ASD individuals have been infrequently investigated, it has been suggested that abnormal dopamine function in the mPFC occurs in these individuals, thus implicating a role of this pathway in cognitive symptoms of ASD (Ernst et al., 1997).

### Abnormalities of the PFC are associated with autism

It might be expected that as a function of loss or dysregulation of cerebellar input to the PFC, that the PFC becomes structurally and/or functionally abnormal in ASD patients. Studies investigating structural abnormalities of the PFC indicate that the frontal cortex is larger in individuals with autism, and the increased volume of frontal cortex has been shown to be positively correlated with autistic symptoms (Carper and Courchesne, 2005; Kumar et al., 2010). Most significantly, the degree of enlargement of the frontal cortex was directly and positively correlated with the degree of hypoplasia in the cerebellum (Carper and Courchesne, 2000). In addition, imaging studies indicate abnormal PFC function in patients with autism. Positron emission tomography (PET) studies demonstrate abnormal mPFC activation during theory of mind tasks and facial expression processing in ASD patients (Castelli et al., 2002; Wang et al., 2007; Wong et al., 2008; Gilbert et al., 2009). Recent fMRI studies also show lower activity levels within the mPFC in individuals with autism during a spatial working memory task (Luna et al., 2002). Furthermore, multi-voxel pattern analyses following fMRI during attentional tasks have shown that the spatial distribution of activation of the mPFC differs between those with and without autism suggesting abnormal functional organization of the mPFC in ASD patients (Gilbert et al., 2009).

The involvement of either the cerebello-ventral tegmental-cortical pathway or the cerebello-thalamocortical pathway in autism would implicate dopaminergic dysfunction, as the cerebellum modulates PFC dopamine release by both of these neuronal circuits. Although to-date this possibility has been sparsely investigated in human patients, there is a small amount of concordant evidence. For example, systemic administration of dopamine antagonists such as haloperidol or risperidone has been shown to reduce aggressive behaviors, social impairments, and stereotypies in ASD patients (Cohen et al., 1980; Campbell et al., 1982; Canitano, 2006). In contrast, administration of bromocriptine, a dopamine agonist, has been shown to have therapeutic effects such as a reduction in hyperactivity and attention deficits for ASD patients (Dollfus et al., 1992). Recent biochemical analysis of cerebrospinal fluid (CSF) levels of dopamine metabolites such as homovanillic acid (HVA) have produced conflicting results, with some studies reporting elevated levels in ASD patients and others reporting no differences between patients and controls (Lam et al., 2006). These disparate systemic drug studies and biochemical findings may not be surprising given that centrally administered dopamine drugs act at diverse sites throughout the brain and CSF levels of neurotransmitter metabolites reflect whole brain neurotransmission activity. A single PET study has demonstrated that children with autism have reduced dopaminergic activity in the mPFC (Ernst et al., 1997). While current evidence of dopamine dysfunction in ASD patients is inconsistent, relatively few studies have investigated dopamine localized to specific brain areas. Therefore, it remains possible that dopaminergic activity within certain brain areas, such as the mPFC, is abnormal in ASD individuals.

### Abnormalities of the thalamus are associated with autism

If the cerebello-thalamocortical circuit is involved in autism, abnormalities of the thalamus should also be expected in ASD patients. Recent MRI studies indirectly corroborate this notion by concluding that while total brain volume is positively correlated with the volume of the thalamus in control brains, a lack of correlation exists between total brain volume and volume of the thalamus in autistic brains (Tsatsanis et al., 2003; Hardan et al., 2006; Tamura et al., 2010). The size of the thalamus in ASD individuals is reduced when compared to controls, and the size of the left thalamus in children with ASD is inversely correlated with stereotypical and repetitive behaviors (Tamura et al., 2010; Estes et al., 2011). Mizuno et al. (2006) also report that the temporal correlation between neurophysiological events in the thalamus and the cortex in ASD individuals exceeds that of controls, as measured by functional connectivity MRI (fcMRI). Additionally, during a visually guided saccade task, ASD individuals have been shown to have greater activation of the medial thalamus as compared to controls (Takarae et al., 2007). The thalamus has also been implicated in cognition in non-autistic individuals as lesions to the thalamus, particularly the mediodorsal nuclei, result in autism-like cognitive deficits such as poor working memory, perseveration, failure to inhibit inappropriate behaviors, and language deficits during recall and recognition tasks (Schmahmann and Pandya, 2008; Gong et al., 2011).

## Rodent models with cerebellar abnormalities

The above-mentioned research clearly implicates the cerebellum in autism (Courchesne et al., 1988). Similarly, the PFC and thalamus have also been implicated in this disorder, which could possibly be explained by downstream effects of cerebellar pathology on cerebello-cortical circuitry. Abnormalities in structure and function of all these areas have been associated with the appearance or severity of autism symptoms (Carper and Courchesne, 2005; Tamura et al., 2010). The use of animal models, which allow for manipulation of genetic and environmental influences, is an effective means of further elucidating both distal and proximal etiological factors and their potential impact on cerebello-cortical circuitry. Some existing rodent models of autism, as well as some models not previously applied to the study of this disorder, display cerebellar, behavioral, and motor abnormalities that parallel those commonly seen in ASD patients. Genetic, viral infection, toxic exposure, and developmental neuropathology models exist that display the signature psychomotor impairments and cerebellar pathology of autism, and are therefore useful for investigating the behavioral impact of changes in cerebello-cortical circuitry. The novel findings produced from research utilizing rodent models could therefore confirm and extend our understanding of the neurochemical and behavioral impact of changes in cerebellar-cortical circuitry.

### Genetic models

As previously discussed, autism is known to be a product of multiple genetic mutations and is highly heritable. Genetic mutations associated with autism, ASD, and similar cognitive disorders have been replicated in rodents. As shown in Table 1, some of these rodent models display both autism-like behavior and cerebellar pathology including the FMR1 knockout (KO) mouse, the engrailed homeobox 2 (EN2) KO mouse, and the staggerer mouse (Goldowitz and Koch, 1986; Petit et al., 1995; Lalonde et al., 1996; Kuemerle et al., 1997; Doulazmi et al., 2001; Rogers et al., 2001; Ellegood et al., 2010). Similarly, the Shank3 gene has been associated with behavioral and cerebellar abnormalities, making Shank3 mutant mice potential candidates for investigations of impaired cerebello-cortical circuitry (Peça et al., 2011; Wang et al., 2011). Also included in this category is the Lurcher (Lc/+) mouse, a mutant with a gain of function in the gene that encodes the Grid2 locus (glutamate receptor, ionotropic, delta2) (Caddy and Biscoe, 1979). Although the Grid2 locus has not been associated with ASD, there is substantial overlap between the morphological and behavioral phenotypes between these mice and patients with these disorders.

**Table 1:** **Summary of genetic rodent models with cerebellar and behavioral abnormalities that parallel those observed in autism**.

**Rodent model**	**Behavioral abnormalities**	**Cerebellar abnormalities**	**Prefrontal cortex abnormalities**
FMR1 KO mice	Hyperactivity	Elongated spines on Purkinje cells	Hyperconnectivity of layer 5 pyramidal cells
	Reduced spatial learning	Decreased volume of deep cerebellar nuclei	Synapses between layer 5 pyramidal cells do not recover from LTD as quickly as controls
	Memory deficits		
	Reduced fear conditioning		
EN2 KO mice	Decreased play	Cerebellar hypoplasia	
	Decreased social behaviors	Decreased Purkinje cell number	
	Reduced aggressive behavior	Foliation defects	
Staggerer mutant mice	Impaired spatial and reversal learning	Cerebellar hypoplasia	
	Memory deficits	Decreased Purkinje cell number	
	Perseverative behavior	Ectopic and atrophic Purkinje cells	
	Abnormal responses to novel environments	Decreased number of granule cells	
		Reduced volume in deep cerebellar nuclei	
Shank3 mutant mice	Social abnormalities		
	Repetitive behavior		
	Learning and memory deficits		
Lurcher mutant mice	Impaired behavioral flexibility	Decreased Purkinje cell number	Decreased mPFC dopamine release following DN stimulation
	Repetitive behavior		

### FMR1 knockout mice

The FMR1 gene codes for the production of the fragile X mental retardation protein known to be involved in cognitive development, and mutations of this gene result in fragile X syndrome (Verkerk et al., 1991; Goodrich-Hunsaker et al., 2011). As discussed previously, patients with fragile X syndrome exhibit autism-like cognitive symptoms and have an increased probability of an autism diagnosis (Rogers et al., 2001; Tsiouris and Brown, 2004). Because of this, FMR1 KO mice have been used to model autism. These mice display several behavioral deficits similar to those seen in autism such as hyperactivity, perseverative behavior, reduced spatial learning abilities, memory deficits, and reduced fear conditioning (Bernardet and Crusio, 2006; Ey et al., 2011; Olmos-Serrano et al., 2011). FMR1 KO mice have cerebellar abnormalities such as elongated spines on cerebellar Purkinje cells and decreased volume of cerebellar nuclei (Koekkoek et al., 2005; Ellegood et al., 2010). Also, eye-blink conditioning, known to be dependent on synaptic plasticity of cerebellar neurons (Kim and Thompson, 1997), is attenuated in FMR1 KO mice suggesting that these cerebellar abnormalities have functional relevance (Koekkoek et al., 2005). Abnormalities also exist in the PFC of FMR1 KO mice. Layer 5 pyramidal neurons display hyperconnectivity in the mPFC, and synapses between these neurons are not able to recover from long term depression as rapidly as the same neurons in control mice (Testa-Silva et al., 2012).

### EN2 knockout mice

The EN2 gene has been associated with autism (Petit et al., 1995; Sen et al., 2010). EN2 is a transcription factor that regulates gene expression necessary for normal cerebellar development. Thus, EN2 KO mice also have cerebellar abnormalities that resemble those observed in autism such as hypoplasia, a reduction in the number of Purkinje cells by approximately 40%, and foliation defects (Millen et al., 1994; Kuemerle et al., 1997). EN2 has also been identified as important for the development and survival of midbrain dopaminergic neurons such as those located in the VTA (Wallén and Perlmann, 2003; Sonnier et al., 2007). EN2 KO mice display social impairments which parallel symptoms of autism including decreased play, social behavior, and aggressive behavior as compared to controls (Cheh et al., 2006).

### Staggerer mutant mice

The staggerer mouse has a mutation of the retinoic acid receptor-related orphan receptor alpha (RORα) gene which has been associated with autism (Hamilton et al., 1996; Nguyen et al., 2010). This gene is responsible for Purkinje cell survival and differentiation (Boukhtouche et al., 2006). The staggerer mouse has cerebellar hypoplasia resulting from a perinatal loss of approximately 80% of Purkinje cells with the remaining Purkinje cells displaying defects such as reduced size and ectopic location (Herrup and Mullen, 1979; Herrup et al., 1996; Doulazmi et al., 2001). Other cerebellar abnormalities such as a near 100% reduction in the number of granule cells and reduced volume of the cerebellar nuclei are also noted in these animals (Landis and Sidman, 1978; Roffler-Tarlov and Herrup, 1981). These mice are ataxic, which limits behavioral research, but have been found to display behavioral deficits including impaired spatial learning and reversal learning, memory deficits, perseverative behavior, and abnormal responses to novel environments (Goldowitz and Koch, 1986; Misslin et al., 1986; Lalonde et al., 1996; Lalonde and Strazielle, 2008).

### Shank3 mutant mice

In humans, the Shank3 gene is found at chromosome 22q13 and codes for a structural protein necessary for the maintenance and plasticity of postsynaptic densities located in excitatory synapses (Boeckers et al., 2002; Durand et al., 2007). An abnormal Shank3 mutation accounts for approximately 2% of autism cases and has been associated with the occurrence of cognitive deficits and disorders with similar cognitive phenotypes such as chromosome 22q13 deletion syndrome (Bonaglia et al., 2006; Betancur et al., 2009; Bozdagi et al., 2010; Chen et al., 2011; Peça et al., 2011). ASD patients with Shank3 deletions are also noted to have severe core symptoms and mental disabilities (Chen et al., 2011). Multiple models exist with Shank3 mutations, and several display behaviors analogous to the core symptoms of autism (Bozdagi et al., 2010; Bangash et al., 2011; Peça et al., 2011; Wang et al., 2011). For example, the isoform-specific Shank3 (e4-9) homozygous mutant exhibits abnormal social behaviors, repetitive behaviors, and learning and memory deficits (Wang et al., 2011). Although research has not yet directly addressed the effect of Shank3 mutations on cerebellar anatomy and function, recent research suggests that mutations of Shank3 may be related to cerebellar abnormalities in both humans and rodents. Shank3 has been shown to be tissue-specific with high expression in the cerebellar granule cells (Beri et al., 2007; Peça et al., 2011). Shank3 has also been suggested to play an important role in the recruitment of axon terminals to cerebellar granule cell dendrites (Roussignol et al., 2005). In addition, mutations of Shank3 are associated with abnormalities or reduced synaptic expression of glutamate receptors (Bangash et al., 2011; Verpelli et al., 2011).

### Lurcher mutant mice

Lurcher mutant mice have an autosomal dominant mutation that results in nearly a complete loss of cerebellar Purkinje cells between the 2nd and 4th weeks of life (Caddy and Biscoe, 1979; Zuo et al., 1997). Since these mice are ataxic and display characteristic lurching movements, chimeric mice, which have a variable loss of Purkinje cells dependent upon the incorporation of the wildtype lineage, have been used to examine the behavioral impact of Purkinje cell loss (Goldowitz et al., 1992). Lurcher mice and chimeras display impaired executive function as measured by a serial reversal learning task (executive function), and learning errors were negatively correlated with the number of Purkinje cells in subsequent cell counts (Dickson et al., 2010). Belzung et al. (2001) found that Lurcher mutant mice showed memory deficits in a radial arm maze. Martin et al. (2010) found a negative correlation between repetitive behaviors and cerebellar Purkinje cell number in chimeric mice. Thus, these mice exhibit executive dysfunction and repetitive behaviors directly related to cerebellar Purkinje cell numbers and approximate the symptoms commonly observed in autism.

Recent research in Lurcher mice has also explored the cerebello-thalamocortical and cerebello-ventral tegmental-cortical circuits. Mittleman et al. (2008) used fixed potential amperometry (FPA) to detect dopamine release in the mPFC following electrical stimulation of the cerebellar Purkinje cell layer or DN of the cerebellum in mice. In Lurcher mice with significant developmental cerebellar Purkinje cell loss, mPFC dopamine release following stimulation at either site was markedly attenuated in comparison to littermate mice with normal Purkinje cell numbers.

Rogers et al. (2011) further examined these two neuronal circuits by using FPA to detect DN stimulation-evoked dopamine release in the mPFC of mice before and after infusions of lidocaine, a local anesthetic, or kynurenate, a broad spectrum glutamate ionotropic receptor antagonist, into the VTA, mediodorsal thalamus, or ventrolateral thalamus. Both lidocaine and kynurenate infusions into the VTA resulted in a comparable decrease in dentate stimulation-evoked mPFC dopamine release (approximately 50%). In a similar fashion, lidocaine or kynurenate infusions into the thalamus resulted in approximately 50% decrease in dentate stimulation-evoked dopamine release in the mPFC suggesting that these pathways equally contribute to mPFC dopamine modulation and that they are primarily, if not entirely, glutamatergic. Although the Lurcher and chimera models have no known genetic overlap with autism, these findings are important because they demonstrate that developmental cerebellar neuropathology in the form of Purkinje cell loss results in autistic-like symptomatology as well as a dysregulation of dopamine release in the mPFC.

**Figure 2:**
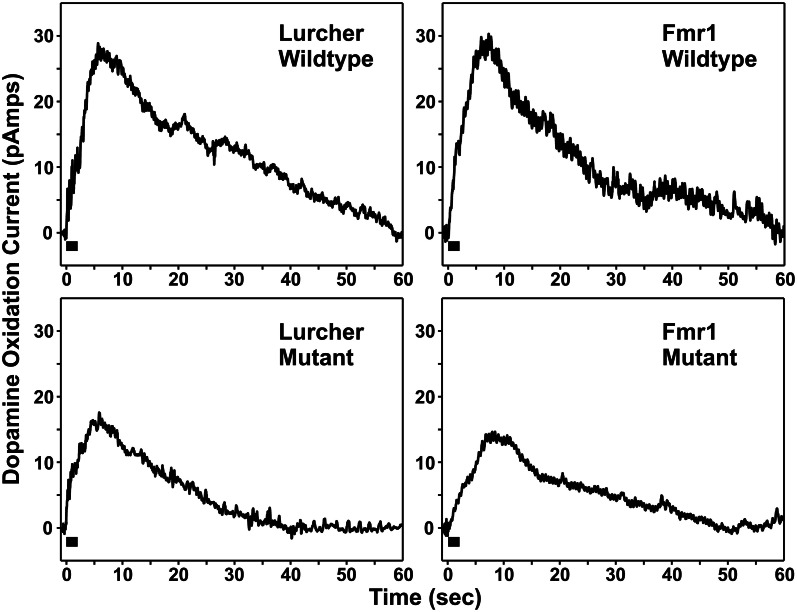
**Individual examples of medial prefrontal cortex dopamine release responses evoked by electrical stimulation (black bar, 100 pulses at 50 Hz) of the dentate nucleus**.

It should be noted that FMR1 mutant mice that have an Fmr1^tm1Cgr^ targeted mutation and Lurcher mutants have recently been compared by Rogers et al. in order to determine if both mutant strains show similar changes in dopamine release in the mPFC following electrical stimulation of the cerebellar DN (Rogers et al., 2013). Similar to Mittleman et al. (2008) FPA was used to monitor mPFC dopamine release evoked by DN electrical stimulation in both mutant strains and their littermate controls. As shown in Figure 2, in comparison to their respective littermate controls, mutants of both strains showed markedly attenuated dopamine release in the mPFC following brief electrical stimulation of the DN, a main output nucleus of the cerebellum. The attenuation in PFC dopamine release was accompanied by a functional reorganization of VTA and thalamic projections. Rogers et al. (2013) observed that in Lurcher and FMR1 wildtype mice, cerebellar modulation of mPFC dopamine was mediated equally by the VTA and thalamic pathways. However, in both the Lurcher mutant and FMR1 mutants, there was a shift in modulatory control away from the VTA toward the thalamic pathway, with a specific increase in modulatory strength of the dopamine signal through the ventrolateral nucleus of the thalamus. These results support the notion that compromised functionality of the projections from the DN to mPFC is a consistent result of developmental damage to the cerebellum and provides an explanation for the observed attenuation of mPFC dopamine release.

### Viral infection models

Children with autism display a variety of inflammatory-like abnormalities in the brain and periphery suggesting autoimmune system activation (Vargas et al., 2005; Careaga et al., 2010; Rose et al., 2012). Viral infections and maternal immune activation have therefore been suggested to play a role in the etiology of autism (Libbey et al., 2005; Patterson, 2009, 2011). Atladóttir et al. (2010) determined that maternal viral infection occurring in the first trimester is associated with the diagnosis of autism. Viral infections of developing nervous systems can alter normal development making the risk of neuropathology following prenatal or neonatal exposure particularly high for areas including the cerebellum and neocortex that continue to develop after birth (Johnson, 1972; Sells et al., 1975). As shown in Table 2, cerebellar and frontal cortex abnormalities following viral infection have been demonstrated in two rodent models, the neonatal Borna disease virus (BDV) infection rat model and the maternal influenza infection mouse model. These two animal models also display autism-like behavioral deficits making them useful for evaluating behavioral changes following developmental cerebellar pathology.

**Table 2:** **Summary of viral infection rodent models with cerebellar and behavioral abnormalities that parallel those observed in autism**.

**Rodent model**	**Behavioral abnormalities**	**Cerebellar abnormalities**	**Prefrontal cortex abnormalities**
Neonatal Borna disease virus infection rats	Hyperactivity	Cerebellar hypoplasia	High levels of viral nucleic acid in PFC
	Stereotypic behavior	Decreased Purkinje cell number	Elevated levels of DOPAC
	Deficits in social behavior		
Maternal influenza infection mice	Reduced exploratory behavior	Atrophy of cerebellum	Gene expression changes in PFC
	Social impairments	Gene expression changes in cerebellum	
	Working memory deficit		
	Impaired emotional behavior	Decreased Purkinje cell number	
		Heterotopic Purkinje cells	
		Delayed migration of granule cells	

### Neonatal Borna disease virus infection rats

BDV is a non-segmented, negative, single-strand virus that replicates in neuronal nuclei of many species and produces a range of neurological symptoms such as motor, sensory, and emotional impairments (Richt et al., 1992; Eisenman et al., 1999; Pletnikov et al., 1999). Although BDV is not considered a potential cause of autism, BDV rodent models have neuroanatomical and behavioral abnormalities representative of the disorder. Rats neonatally exposed to BDV display several behavioral impairments including hyperactivity, impaired spatial learning, memory deficits, decreased open-field exploration, stereotypic behavior, and deficits in social behavior (Hornig et al., 1999; Pletnikov et al., 1999; Rubin et al., 1999). Neonatal BDV infection in rats produces cerebellar hypoplasia and has a relatively high specificity for Purkinje cells resulting in up to 75% loss of these cells (Bautista et al., 1995; Eisenman et al., 1999; Hornig et al., 1999; Zocher et al., 2000; Pletnikov et al., 2003; Williams and Lipkin, 2006). Solbrig et al. (1996) employed high performance liquid chromatography (HPLC) to investigate the PFC of infected rats and found high levels of viral nucleic acid and elevated levels of the dopamine metabolite 3,4-dihydroxy-phenylacetic acid (DOPAC) in the PFC suggesting a role for PFC dopamine hyperactivity in the behavioral changes observed in this model.

### Maternal influenza infection mice

In a sample of mothers hospitalized during pregnancy, 50% of those whose offspring was diagnosed with autism were hospitalized due to influenza. Also, according to this study, the frequency of autism diagnosis among children of mothers exposed to influenza during the first trimester was approximately 6 times higher than in the general population (Atladóttir et al., 2010). Similarly, rodents prenatally or neonatally exposed to influenza virus display a range of behavioral deficits including social impairments and cognitive deficits, such as impaired working memory and impaired emotional behavior (Shi et al., 2003; Beraki et al., 2005; Asp et al., 2009). Atrophy of the cerebellum and gene expression changes in the PFC and cerebellum have also been observed in mice prenatally exposed to influenza virus (Fatemi et al., 2008, 2009). Most significantly, prenatal exposure to influenza in mice results in approximately 33% decrease in Purkinje cell numbers, heterotopic Purkinje cells, and delayed migration of granule cells in the cerebellum (Shi et al., 2009). Also, Fatemi et al. (2004) observed an increase in GAD 67-kDa protein, a rate limiting enzyme that catalyzes synthesis of GABA from L-glutamate, in mice exposed to influenza and suggested that this was evidence of aberrant glutamatergic activity.

### Toxic exposure model

Environmental factors such as exposure to toxic chemicals during pregnancy are also thought to contribute to autism. Thus, some rodent models utilize prenatal or neonatal exposure to teratogens known to be associated with autism. As shown in Table 3, one such model is the prenatal valproic acid exposure rat model which displays signature behavioral and cerebellar abnormalities that parallel those observed in autism (Wagner et al., 2006).

**Table 3:** **Summary of toxic exposure rodent model with cerebellar and behavioral abnormalities that parallel those observed in autism**.

**Rodent model**	**Behavioral abnormalities**	**Cerebellar abnormalities**	**Prefrontal cortex abnormalities**
Prenatal valproic acid exposure rats	Deficits in olfactory discrimination	Apoptotic cells in cerebellum	Aberrant dopamine activity in PFC
	Decreased prepulse inhibition	Decreased Purkinje cell number	
	Hyperactivity		
	Increased stereotypy		
	Decreased social play		
	Decreased exploratory behavior		

### Prenatal valproic acid exposure rats

VPA is an antiepileptic drug found to be positively correlated with the occurrence of autism in the offspring of mothers exposed to the drug during pregnancy (Arndt et al., 2005). In humans, the behavior resulting from prenatal exposure includes delayed speech and motor development, hyperactivity, poor social and communication skills, and deficits in cognitive functions such as attention (Moore et al., 2000). Likewise, mice and rats prenatally exposed to VPA exhibit abnormal behaviors including deficits in olfactory discrimination, decreased prepulse inhibition, hyperactivity, increased stereotypy, decreased social play, and decreased exploratory behavior, as compared to controls (Schneider and Przewlocki, 2005; Roullet et al., 2010). Rats prenatally exposed to VPA also exhibit cerebellar abnormalities. Yochum et al. (2008) found that prenatal exposure to VPA in rats induced an increase in apoptotic cells in the cerebellum, while Ingram et al. (2000) found that rats prenatally exposed to a single VPA intra-peritoneal injection had approximately 30% fewer Purkinje cells and significantly differed from control animals. Consistent with the disconnection hypothesis, VPA seems to have an effect on dopamine tissue levels in adult rodents following acute or chronic exposure (Löscher, 1999). Adult rats that received chronic i.p. injections of VPA had increased dopamine tissue levels in multiple brain areas, including frontal cortex, as measured by HPLC (Baf et al., 1994). Also, an *in vivo* microdialysis study found that an acute subcutaneous injection of VPA produced increased extracellular dopamine levels in the mPFC of rats (Ichikawa and Meltzer, 1999).

## Summary and conclusions

In attempting to answer the question, “Is autism a disease of the cerebellum,” it is apparent from clinical research that developmental damage to the cerebellum is a common occurrence in ASD. Pre-clinical research confirms clinical observations that there are multiple routes to developmental cerebellar damage including genetic mutations, viral exposure, or exposure to environmental toxins. Rodent models of autism both confirm often reported clinical findings and also extend these findings by providing candidate pathways deserving of further research, although both types of research are potentially limited by questions about the selectivity of cerebellar abnormalities as opposed to the rest of the brain. It is however intriguing to note that developmental damage to the cerebellum is frequently associated with a diagnosis of ASD in humans, and in rodents cerebellar neuropathology is associated with many autism-like cognitive deficits. Thus, a parsimonious answer to this initial question is, “Yes,” in many cases it is likely that autism is associated with developmental cerebellar damage.

Is developmental cerebellar damage the only route to the symptoms of autism? We believe that the most likely answer to this question is “No,” simply because the heterogeneity of symptoms as well as the range of symptom severity from savant skills to profound mental and physical handicaps makes it unlikely that there is a unitary cause for these disorders.

A third question that also should be addressed is, can glutamatergic projections to the PFC be affected independently of developmental cerebellar damage? Pre-clinical research has demonstrated that two glutamatergic pathways from the cerebellum to the PFC can modulate dopamine release in the mPFC. This is significant because impairments in attention, memory, and a marked tendency to perseverate in the face of changing contingencies (i.e., executive function) are well documented in patients with ASD, and are well known to be dependent on on-going dopaminergic activity in the mPFC (Seamans and Yang, 2004). Research in lurcher and FMR1 mutant mice has shown that the ability of the cerebellum to modulate mPFC dopamine release is significantly affected by developmental cerebellar damage involving Purkinje cells, and in Lurchers, that cerebellar Purkinje cell number is significantly correlated with autism-like deficits in executive function, working memory and repetitive behavior. Thus it is possible that cerebellar neuropathology which results in changes in cerebellar output and subsequent effects on downstream circuitry might underlie some of the cognitive symptoms of autism.

In terms of this question we currently believe that, depending on the severity of developmental cerebellar neuropathology, it is likely that glutamatergic projections to the PFC will be effected and this will result in autistic symptomatology. This may seem surprising given that it has been effectively argued that in a mouse model of Rett syndrome, mutation of the MECP2 gene results in the appearance of autism-like symptoms including increased repetitive behaviors which can be linked to dysfunctions in GABA signaling (Chao et al., 2010). It should be noted, however, that paralleling the time course in the development of symptoms, mutations involving the MECP2 locus also cause progressive degenerative changes in the cerebellum (Murakami et al., 1992; Reardon et al., 2010), as well as altering glutamate homeostasis (Zhong et al., 2012).

In support of the notion that cerebellar pathology and glutamatergic dysregulation co-occur, in a mouse model of fragile X, neuroligin-3 KO mice were found to exhibit deficits in social behaviors analogous to those seen in autism (Baudouin et al., 2012). These mice showed cerebellar pathology involving increases in the metabotropic glutamate receptor mGLUR1α which interfered with the structure and function of circuitry in the cerebellum. Reactivation of the production of neuroligin-3 ameliorated behavioral deficits in these mice and reversed the cerebellar structural deficits that were previously observed.

Our goal in the present manuscript was to provide a galvanizing theory of what may be a major neurophysiological mechanism that could account for the cognitive, motor and reward symptoms of autism, as well as the apparent variability in symptom severity. Thus, acceptance of the idea that developmental damage to the cerebellum is a key player in the etiology of autism and ASDs may have explanatory power in realms other than cognitive symptomatology. As noted previously, the diagnosis of autism is frequently associated with co-occurring deficits in motor function (Gowen and Hamilton, 2013). Given the relationship between cerebellar Purkinje cell loss and the occurrence of ataxia as well as the cerebellar connections to premotor and motor cortical regions (Strick et al., 2009; Fatemi et al., 2012) it seems reasonable to suggest that variability in the degree of cerebellar damage is likely related to the severity of motor deficits in ASD individuals.

With regard to underlying mechanisms of the social reward impairment in autism, preclinical research links dysregulation of meso-accumbens dopamine to deficits in social engagement and social bond formation (Dichter et al., 2012c). As there is now clear evidence that developmental cerebellar damage is consistently associated with dysregulation of mesocortical dopamine projections arising from the VTA in Lurcher and FMR1 mice (Rogers et al., 2013), it is highly likely that the meso-accumbens dopaminergic projection, also arising from the VTA, may also show deficits in neurotransmission. As the meso-accumbens system has been heavily implicated in reward and reinforcement (e.g., Salamone and Correa, 2012) it is reasonable to suggest that a putative dysfunction in meso-accumbens dopamine, that is driven by developmental cerebellar neuropathology, could account for the deficits in reward responsivity displayed by ASD patients.

While it seems clear that cerebellar modulation of mPFC dopamine release is dependent on glutamatergic pathways, there remains a significant gap in our knowledge of the role of dopaminergic and glutamatergic systems in autism. Several molecular biological techniques are available for investigating this in rodents, including the use of viral vectors to selectively transduce, or introduce new genetic material into glutamatergic neurons. This might provide a way to understand the behavioral consequences and ameliorate behavioral impairments associated with altered glutamatergic transmission within this circuitry. Future research should also aim to determine when during the developmental time course cerebellar lesions occur as well as the anatomical specificity of the cerebellar deficits observed in the reviewed rodent models. Understanding the developmental timing and the anatomical specificity of cerebellar pathology is important for understanding the causal mechanisms underlying this consistent feature of autism. Additionally, given the apparent glutamatergic dysregulation in the cerebello-ventral tegmental-cortical pathway that occurs along with developmental cerebellar damage we believe the scope of research should be expanded to include investigations of other brain regions that receive efferents from nuclei in this circuit, and especially the PPT. It is well known that projections from the PPT to the substantia nigra modulate dopamine transmission in the dorsal striatum (Forster and Blaha, 2003). Thus, it would be expected that developmental cerebellar damage is also associated with dysregulation in striatal dopamine transmission. Ultimately, a better understanding of the direct effects of cerebellar pathology in autism could lead to better diagnostic measures, earlier diagnosis, and novel pharmacological targets for the treatment of autism and ASD.

### Conflict of interest statement

The authors declare that the research was conducted in the absence of any commercial or financial relationships that could be construed as a potential conflict of interest.
